# Supplementary material for: A Super Anticorrosive Ultrathin Film by Restarting the Native Passive Film on 316L Stainless Steel
Source: Nanomaterials (Basel). 2023 Jan 16;13(2):367. doi: 10.3390/nano13020367 (PMC9862606; doi:10.3390/nano13020367)
Supplement: Supplementary file 1 [file nanomaterials-13-00367-s001.zip › nanomaterials-2116941-supplementary.pdf]

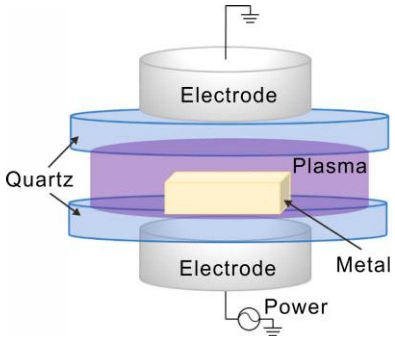

Figure S1. The schematic of dielectric barrier discharge for surface treatment of metals.

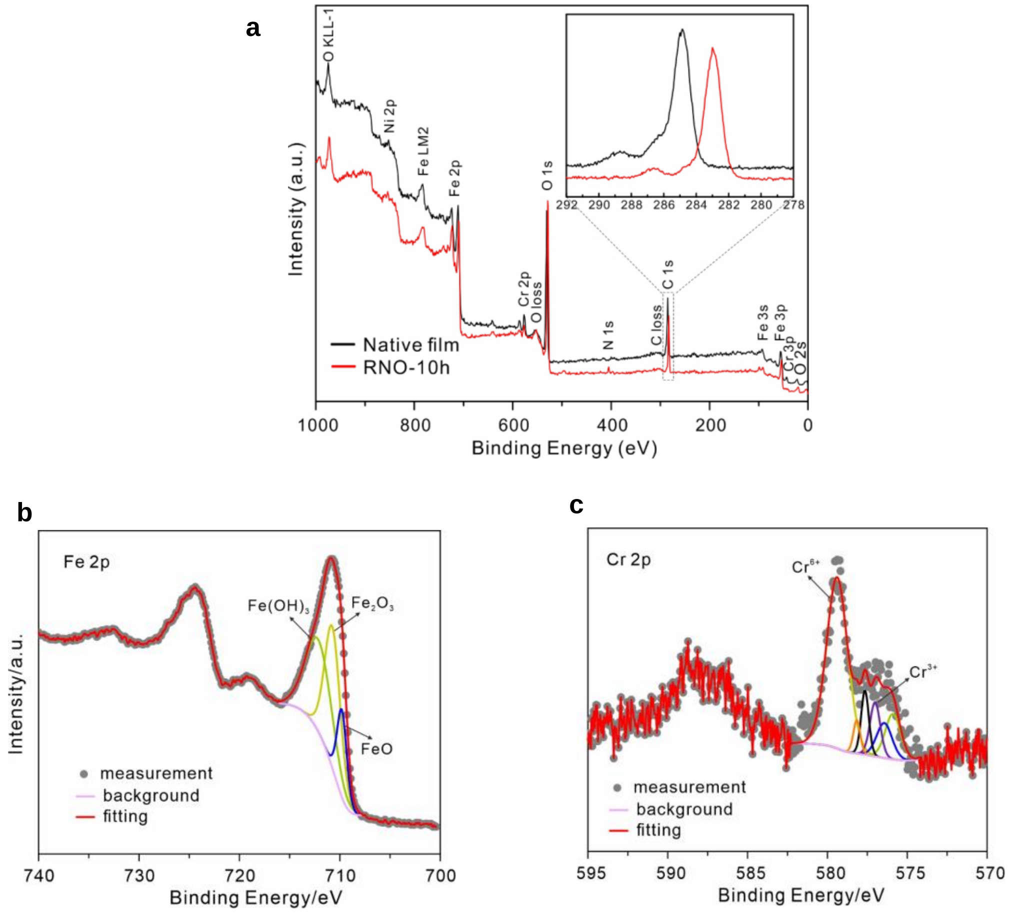

Figure S2. (**a**) XPS survey spectra of 316L SS and the RNO-10h 316L SS. The inset spectra of C 1s showed the negative shift of 1.9 eV for the whole spectra of RNO-10h 316L SS. It was indicated that the surface was negatively charged. (b), The RNO-10h sample was calibrated by standard C 1s, and the fitting results of Fe 2p3/2 spectrum indicated that the film consisted of $Fe(OH)_3$, Fe_2O_3 and FeO. (**c**), The Cr 2p3/2 spectrum was fitted using one peak at 579 eV for Cr^{6+}, and a series of five peaks for Cr^{3+} owing to multiplet splitting. The metallic Cr and Fe have disappeared. [13,15].

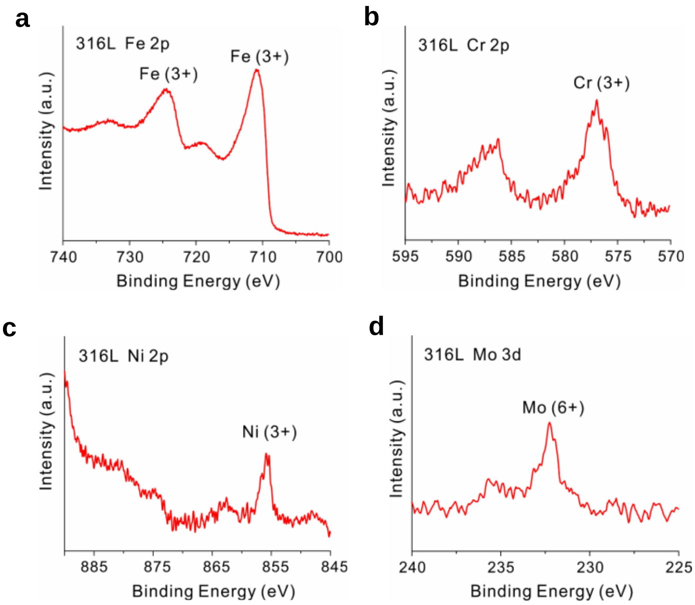

Figure S3. XPS spectra of the RNO-10h after 3 d of immersion in 0.6 M NaCl solution at room temperature. (**a**), Fe^{3+} did not change during the immersion. (**b**), Cr^{6+} had been reduced to Cr^{3+}. (c), Ni^{3+} in film did not change during the immersion. (**d**), Mo^{6+} did not change during the immersion.

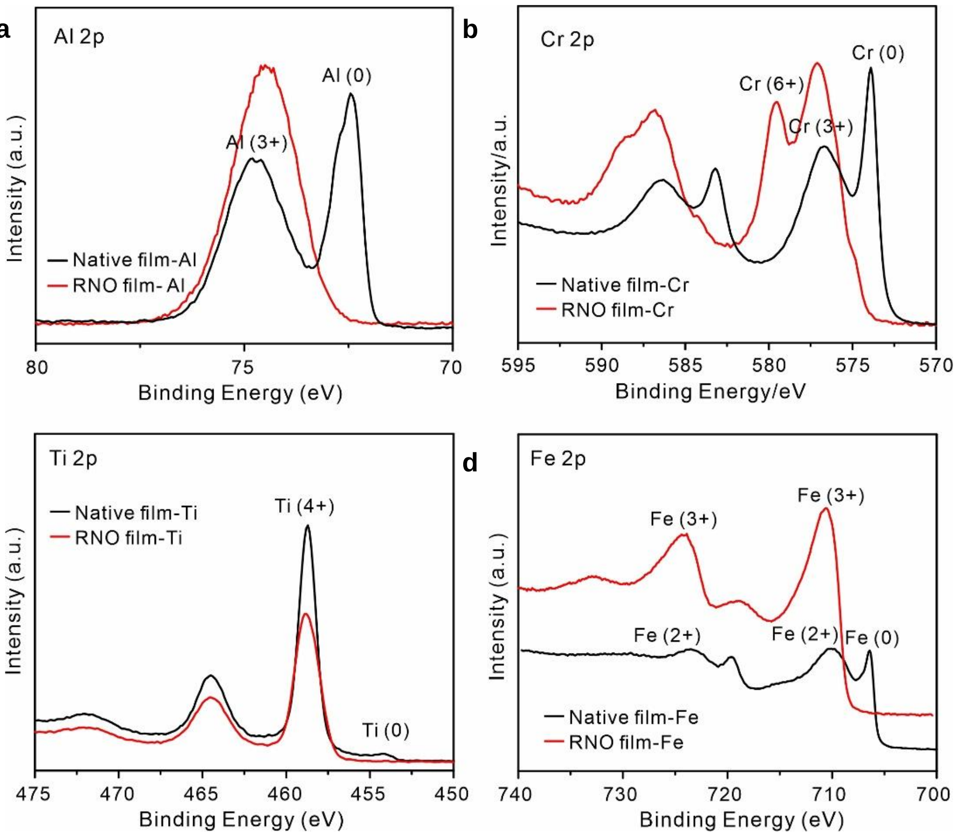

Figure S4. XPS spectra of pure metal surfaces before (black) and after (red) 1 h of processing, indicating the generality of the processing in metal surface oxidation. (**a**), the disappearing of metallic Al indicates the thickening of the oxide film. (b), the disappearing of metallic Cr indicates the thickening of the oxide film on Cr. (**c**), the disappearing of metallic Ti indicates the thickening of the oxide film on Ti. (**d**), the disappearing of metallic Fe indicates the thickening of the oxide film on Fe.

Table 1. EIS parameters extracted by fitting EIS spectra (Figure 3d).

**Samples**	**R_s ($\Omega\cdot cm^2$)**	**C_f ($10^{-5}\cdot\Omega^{-1}\cdot s^{-n}\cdot cm^{-2}$)**	**n_1**	**R_f (10^5 $\Omega\cdot cm^2$)**	**C_{dl} ($10^{-5}\cdot\Omega^{-1}\cdot s^{-n}\cdot cm^{-2}$)**	**n_2**	**R_{ct} ($10^5\cdot\Omega\cdot cm^2$)**
Bare 316L SS	2.32	–	–	–	6.24	0.91	2.06
RNO-10 min	2.69	–	–	–	2.81	0.89	32.5
RNO-1h	3.17	0.83	0.84	70.1	0.62	0.72	0.029
RNO-10h	5.47	0.85	0.85	48.6	0.35	0.64	1.77

References

[13] Greczynski, G.; Hultman, L. X-ray photoelectron spectroscopy: Towards reliable binding energy referencing. *Prog. Mater. Sci.* 2020, 107, 100591.

[15] Wang Z.; Di-Franco Francesco.; Seyeux A.; Zanna S.; Maurice V.; Marcus P. Passivation-induced physicochemical alterations of the native surface oxide film on 316L austenitic stainless steel, *J. Electrochem. Soc.* **2019**, 166, C3376-C3388.
